# Details of the cooperative binding of piperlongumine with rat serum albumin obtained by spectroscopic and computational analyses

**DOI:** 10.1038/s41598-019-52187-5

**Published:** 2019-10-30

**Authors:** Ana Paula Ribeiro Povinelli, Gabriel Zazeri, Marcelo de Freitas Lima, Marinônio Lopes Cornélio

**Affiliations:** 1 Departamento de Física, Instituto de Biociências, Letras e Ciências Exatas (IBILCE), UNESP, Rua Cristovão Colombo 2265, CEP 15054-000 São José do Rio Preto, SP Brazil; 2 Departamento de Química, Instituto de Biociências, Letras e Ciências Exatas (IBILCE), UNESP, Rua Cristovão Colombo 2265, CEP 15054-000 São José do Rio Preto, SP Brazil

**Keywords:** Biological physics, Molecular biophysics

## Abstract

Piperlongumine (PPL) has presented a variety of important pharmacological activities. In recent pharmacokinetics studies in rats, this molecule reached 76.39% of bioavailability. Although PPL is present in the bloodstream, no information is found on the interaction between PPL and rat serum albumin (RSA), the most abundant protein with the function of transporting endo/exogenous molecules. In this sense, the present study elucidated the mechanism of interaction between PPL and RSA, using in conjunction spectroscopic and computational techniques. This paper shows the importance of applying inner filter correction over the entire fluorescence spectrum prior to any conclusion regarding changes in the polarity of the fluorophore microenvironment, also demonstrates the convergence of the results obtained from the treatment of fluorescence data using the area below the spectrum curve and the intensity in a single wavelength. Thermodynamic parameters revealed that PPL binds to RSA spontaneously (ΔG < 0) and the process is entropically driven. Interaction density function method (IDF) indicated that PPL accessed two cooperative sites in RSA, with moderate binding constants (2.3 × 10^5^ M^−1^ and 1.3 × 10^5^ M^−1^). The molecular docking described the microenvironment of the interaction sites, rich in apolar residues. The stability of the RSA-PPL complex was checked by molecular dynamics.

## Introduction

Piperlongumine, also known as piplartine, is an amide alkaloid isolated from Long pepper^1^ that is widely used in Indian traditional medicine. This natural compound has been calling attention of scientific community because of its biological activities such as antitumor, antimetastatic, antiplatelet aggregation and anxiolytic^2^. Piperlongumine (PPL) is a lipophilic molecule, with logP = 1.63 calculated through the software Marvin (ChemAxon, Budapest, Hungary). This molecule is characterized by the presence of electrophilic frames^3^ and α,β-unsaturated amide as Michael acceptor^4^. In addition, piperlongumine has no Lipinski’s and Lead-like rule violations^5^, which makes this molecule an excellent candidate to be launched as a drug. Published pharmacokinetic studies report that oral, intravenous and intraperitoneal administration in rats results in different times of PPL permanence in plasma^2^. Considering the lipophilic characteristics of PPL, its transport through plasma must be assisted by carrier proteins, such as albumin. A few works present in the literature bring the first results of the interaction of PPL with human albumin^6,7^. Nevertheless, no information about the interaction between PPL and rat serum albumin (RSA) is known. RSA has 584 amino acids with just one tryptophan residue^8^, which enables the use of fluorescence spectroscopy^9^. The crystallographic structure of RSA has not yet been determined. However, RSA modeling has been performed and published in a recent article^10^. Therefore, based on the modeled RSA, it can be compared with the HSA structure deposited in PDB (1AO6) and several similarities can be found between them. The comparison presents that RSA has 73% of sequence similarity (Fig. S1), and high preservation of its tertiary structure (Fig. S2). These proteins share the same number of disulfide bridges (17) and a Cys34 thiol group^11^.

The goal of present investigation is to elucidate the molecular biophysical mechanisms of the interaction between RSA and PPL. Two approach lines of the molecular biophysical field were applied, one experimental another computational. The thermodynamic of the interaction between RSA and PPL is disclosed based on fluorescence spectroscopy analyses. Here, two different data analyses were used to treat fluorescence spectra: the most common approach based on the intensity of the fluorescence signal at one specific wavelength and another based on the areas below the fluorescence spectra. The importance of applying the inner filter correction in all the wavelengths of the spectrum is also discussed. In addition, using fluorescence spectroscopy, the number of sites accessed by PPL was determined through interaction density function (IDF) method. The microenvironment of each site was described using molecular docking and the stability of the complex was verified by means of molecular dynamics.

## Results and Discussion

### Fluorescence analyses

**Figure 1:**
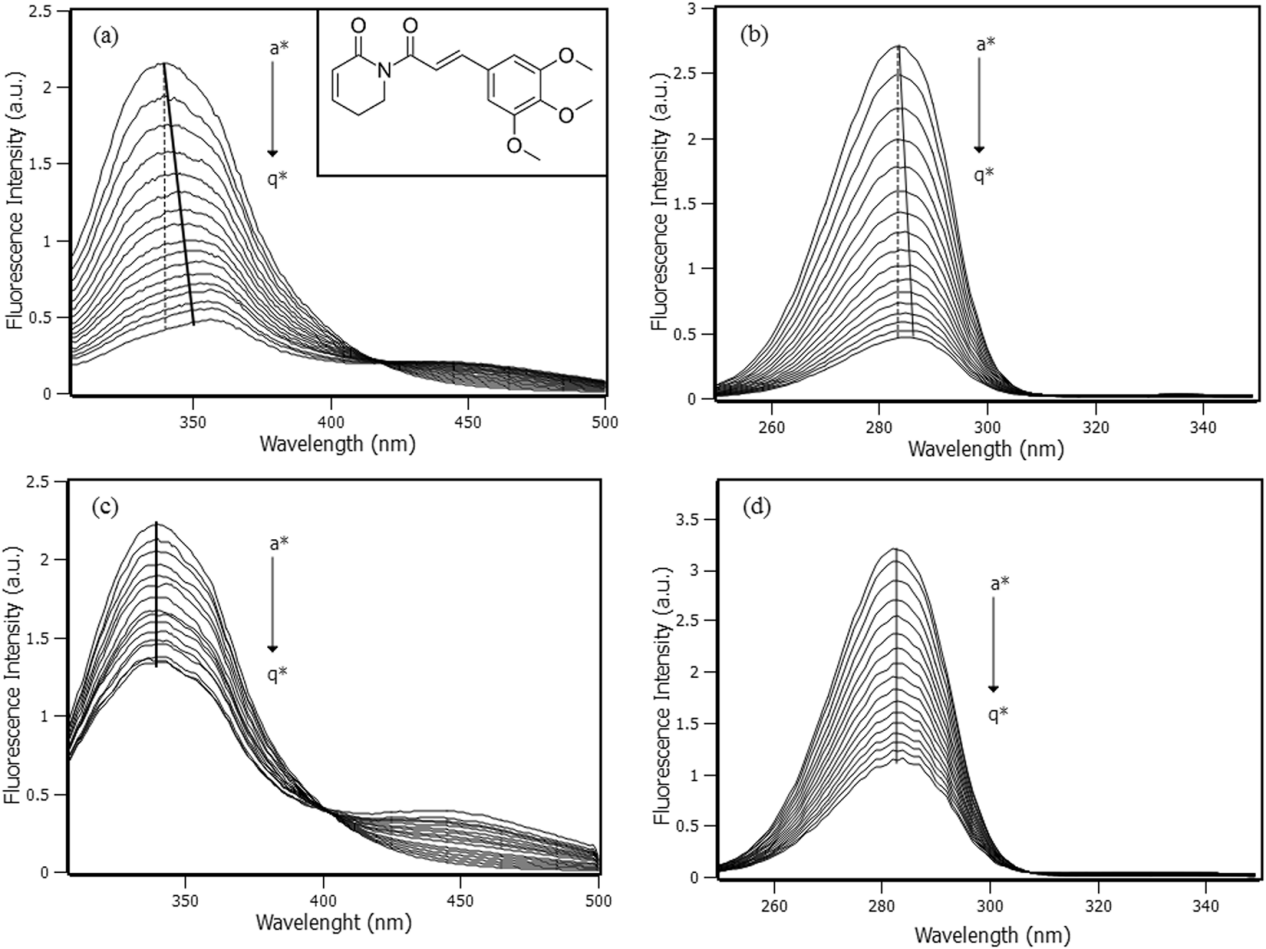
(**a**) Fluorescence emission spectra of RSA with increments of 2 µM of PPL (a* → q*: from 0 to 32 µM), [RSA] = 4 µM, T = 298 K and λ_exc_ = 295 nm. The dashed line marks the intensity at 340 nm, while the solid line marks the maximum intensity shift. The inset shows the PPL structure. (**b**) Synchronous spectra of RSA with increments of 2 µM of PPL (a* → q*: from 0 to 32 µM), [RSA] = 4 µM, T = 298 K, λ_exc_ = 240 nm–350 nm and ∆λ = 60 nm. The dashed line marks the emission intensity at λ_exc_ = 283 nm, while the solid line marks the maximum emission intensity shift. (**c**) Fluorescence emission spectra with inner filter correction, where the solid line marks the maximum intensity at 340 nm. (**d**) Synchronous spectra with inner filter correction, where the solid line marks the maximum emission intensity at λ_exc_ = 283 nm.

The intrinsic fluorescence property of Trp214 was used to comprehend the interaction between PPL and RSA. Figure 1a shows the spectra of Trp214 fluorescence emission under the effect of PPL addition, which caused a decrease on the intensity of Trp214 fluorescence signal and also an apparent shift at the maximum fluorescence to higher wavelengths, commonly named red shift. The same behavior was noticed in synchronous fluorescence spectra (Fig. 1b). Shifts of maximum intensity in both fluorescence emission and synchronous spectra are very common to happen and have an important physical meaning, usually interpreted as a result of local or global protein conformational changes that induce alterations in the polarity of the microenvironment where the fluorophore is located. Red shift is an effect of the exposed fluorophores to a more hydrophilic environment, while blue shift is an effect of the exposed fluorophores to a more hydrophobic environment^12^. Nevertheless, in this work it was verified that in the case in which ligand absorption spectrum overlaps to some degree the fluorophore emission spectrum, the shifts may be only a false positive caused by the inner filter effect. Figure 1c shows the emission spectra with inner filter correction for λ_exc_ = 295 nm and λ_em_ = 305–500 nm, keeping the maximum intensity at 340 nm. Figure 1d shows the synchronous fluorescence spectra with inner filter correction for λ_exc_ = 240–350 nm and λ_em_ = 300–410 nm, keeping the maximum intensity at the excitation wavelength of 283 nm. The application of inner filter correction in both fluorescence emission and synchronous spectra eliminated the apparent red shift observed at Fig. 1a,c and therefore the hypothesis of changing in polarity of Trp214 microenvironment was not confirmed.

How is it possible for inner filter to cause such a shift on the maximum intensity of the spectra? This is due to two concurrent events that occur in the sample. The first requires that some overlap exists between the absorption spectrum of the ligand and the emission spectrum of fluorophore. The second consists of some asymmetry of the profile of the line shape of the absorption spectrum of the ligand in relation to the maximum emission of fluorophore spectrum (see Fig. S3).

Quenching mechanism can be defined as either static (ground state complex) or dynamic (diffusive encounters) process. The most common method reported in literature to determine the quenching mechanism is by comparing Stern-Volmer constants (K_SV_) at different temperatures^13^. The analysis of the bimolecular quenching rate constant (k_q_) is another method that can also be used to indicate the quenching mechanism. In general terms, for collisional quenching process k_q_ cannot be larger than 10^10^ M^−1^s^−1^ ^14^. Usually, K_SV_ is obtained from plots of the ratio of fluorescence intensities at the maximum emission wavelength ($\frac{{F}_{0}}{F}$) versus the ligand concentration ([L]) (Eq. (1)). In this article, the ratio between the areas below the fluorescence spectral curve ($\frac{{A}_{0}}{A}$) was introduced in order to compare and confirm the possibility of its application. However, it is a *sine qua non* condition that the entire spectrum has been previously corrected to avoid the inner filter effect.

1 $$\frac{{F}_{0}}{F}=1+{K}_{SV}[L]=\,1+{k}_{q}{\tau }_{0}[L]\,$$

**Figure 2:**
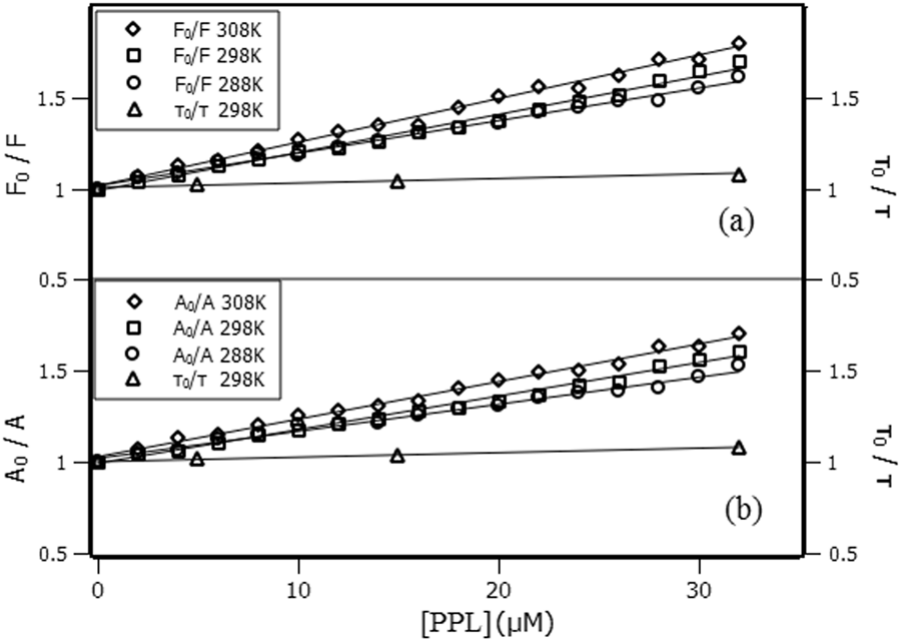
Stern-Volmer plots at 288K, 298K and 308K. (**a**) Ratio of fluorescence intensities at 340 nm. (**b**) Ratio of the areas below the fluorescence spectrum (from 305 to 400 nm). The right ordinate is the variations of Trp lifetime with PPL concentration.

Figure 2a,b show the Stern-Volmer plots at 288K, 298K and 308K with linear responses versus the increment of PPL concentration comparing both approaches intensity and area, respectively. These results indicate that there is a single class of fluorophore in the protein, all equally accessible to the quencher and therefore only one quenching mechanism occurred^15^. Moreover, Stern-Volmer plots show that K_SV_ constant increased with the rise in temperature (Table 1). This result is not conclusive to state that the quenching mechanism is dynamic or static. Therefore, the use of time-resolved fluorescence technique and the analysis of k_q_ are required to actually determine which the mechanism is. The quenching is a result of collisions if there is equivalence between the decrements of Trp214 lifetime and the fluorescence intensity, i.e., τ_0_/τ = F_0_/F. On the other hand, if τ_0_/τ is nearly unaffected and remains close to the unity, the quenching mechanism is static^14^.

**Table 1:** Stern-Volmer constant (K_SV_), binding constant (K_a_) and number of sites (n) calculated through the fluorescence intensities at 340 nm and through the area below the fluorescence spectra in the range 305–400 nm.

Temperature (K)	K_SV_(x10^4^ M^−1^)		k_q_(x10^12^ M^−1^s^−1^)		K_a_ (x10^4^ M^−1^)		n	
	Intensity	Areas	Intensity	Areas	Intensity	Areas	Intensity	Areas
288	1.78 ± 0.04	1.49 ± 0.04	2.65 ± 0.01	2.22 ± 0.01	1.72 ± 0.07	1.49 ± 0.04	0.83	0.86
298	2.11 ± 0.05	1.83 ± 0.04	3.14 ± 0.01	2.72 ± 0.01	2.24 ± 0.09	1.84 ± 0.07	1.02	0.97
308	2.38 ± 0.05	2.08 ± 0.05	3.54 ± 0.01	3.09 ± 0.01	2.54 ± 0.09	2.17 ± 0.09	0.88	0.80

Time-resolved fluorescence indicated that there is not equivalence between τ_0_/τ and F_0_/F (right ordinate of Fig. 2a,b). Furthermore, Table 1 shows that k_q_ is ~10^12^ M^−1^s^−1^, which is two orders of magnitude greater than the quenching rate constant admitted for collisional process. These results lead to the conclusion that collisional quenching is not the main cause of the decrease in fluorescence intensity of Trp214. Besides that, Fig. S4 and Table S1 show a decrease in Trp214 lifetime from 6.71 to 6.28 ns in the 1:8 protein-ligand ratio. Such decrease may be an indicative to some collisional contribution to quenching process. Nevertheless, if this contribution were as significant as the static quenching, Stern-Volmer plot would present the behavior of an upward curvature^14,16^, which was not observed in this study. Therefore, the quenching is dominated by the static process.

The binding constant (K_a_) concerning the interaction between RSA and PPL was calculated through the binding equilibrium model^17^ using the double-log equation (Eq. (2)), where n is the number of sites (Fig. S5).

2 $$\log (\frac{{F}_{0}-F}{F})=n\cdot \,\log \,{K}_{a}-n\cdot \,\log (\frac{1}{[PPL]-(\frac{{F}_{0}-F}{{F}_{0}})\cdot [RSA]})$$

Table 1 shows that K_a_ calculated using the fluorescence intensities at 340 nm and the areas below the spectra are in good agreement. The order of magnitude of K_a_ is 10^4^ M^−1^, that according to literature corresponds to a moderate constant for albumins^7^. The number of sites (n) remained close to one, which is expected due to the first order binding equilibrium model, and the increment of K_a_ as the temperature rises, indicates its influence on the stability of the complex. According to binding constant values, the complex strengthened its interaction with the rise in temperature.

### Thermodynamic parameters

Thermodynamic parameters, such as Gibbs free energy variation (∆G), enthalpy variation (∆H) and entropy variation (∆S), are important clues to interpret the intermolecular forces involved in the interaction between small ligands and proteins. The thermodynamic parameters mentioned are related to each other through Eq. (3), where $\Delta G=\,-\,RT\,\mathrm{ln}\,{K}_{a}$. Therefore Eq. (3) can be rewritten as Van’t Hoff equation (Eq.(4)). The linear regression analysis of Van’t Hoff plot ($\mathrm{ln}\,{K}_{a}$ versus $\frac{1}{T}$) provides ∆H and ∆S obtained from slope and y-intercept, respectively.

3 $$\Delta G=\Delta H-T\Delta S$$

4 $$\mathrm{ln}\,{K}_{a}=-\frac{\Delta H}{R.T}+\frac{\Delta S}{R}$$

**Figure 3:**
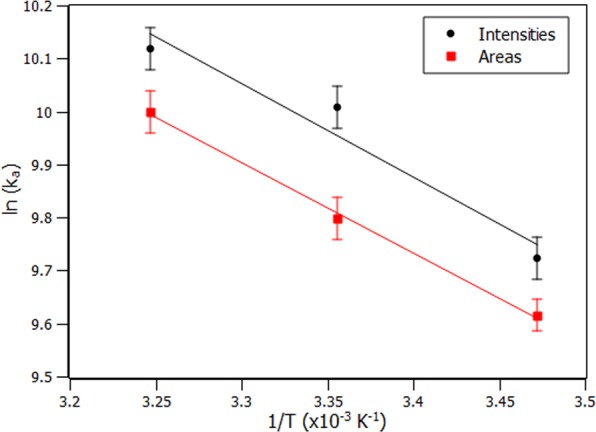
Van’t Hoff plots obtained from intensities (black circles) and from the areas (red squares). Solid lines represent the linear adjustment.

Figure 3 shows the parallelism between Van’t Hoff plot obtained at a single wavelength of the fluorescence spectrum and using the area below the fluorescence spectrum in the range of 305–400 nm.

Table 2 shows the thermodynamic values obtained through the intensity and area that are in good agreement. The area approach showed to be more accurate considering the smaller error range. ∆G exhibited negative values in all the three temperatures confirming a spontaneous process. Table 2 also presents the energy balance having the greater weight in the contribution due to entropy instead of enthalpy; such results indicate that the physical forces present during the interaction may be from hydrophobic nature^18^.

**Table 2:** Thermodynamic parameters (∆G, ∆H and ∆S) of the interaction between RSA and PPL and the comparison of the results obtained using intensities and areas.

T (K)	∆G^o^ (kJ/mol)		∆H^o^ (kJ/mol)		∆S°(J/mol.K)		T. ∆S°(kJ/mol)	
	Intens.	Areas	Intens.	Areas	Intens.	Areas	Intens.	Areas
288	−23.34 ± 0.97	−22.99 ± 0.64	14.64 ± 3.42	14.10 ± 0.66	131.85 ± 11.46	128.81 ± 2.24	37.97 ± 3.30	37.09 ± 0.65
298	−24.80 ± 0.99	−24.32 ± 0.94	14.64 ± 3.42	14.10 ± 0.66	131.85 ± 11.46	128.81 ± 2.24	39.29 ± 3.41	38.38 ± 0.67
308	−25.96 ± 0.91	−25.56 ± 1.00	14.64 ± 3.42	14.10 ± 0.66	131.85 ± 11.46	128.81 ± 2.24	40.61 ± 3.53	39.67 ± 0.69

### Interaction density function (IDF)

Interaction density function is a methodology used to treat experimental data, but differently from binding equilibrium model, IDF does not make use of any model *a priori*^19^. The advantage of applying IDF is the possibility of not only determining the number of binding sites but also identifying cooperativity occurrence among them. IDF considers that, if the free ligand concentration ([PPL]_free_) is the same for two or more solutions at different concentrations of total protein ([RSA]), the average interaction density (Συ_i_) will also be the same and consequently the system will have the same variation on the percentage of quenching (ΔF). The percentage of fluorescence quenching is given by Eq. (5). Where F_obs_ is the observed fluorescence signal in the presence of PPL and F_F_P_T_ is the observed fluorescence signal for free protein.

5 $$\Delta F=\frac{|{F}_{obs}-{F}_{F}{P}_{T}|}{{F}_{F}{P}_{T}}\cdot 100 \% $$

**Figure 4:**
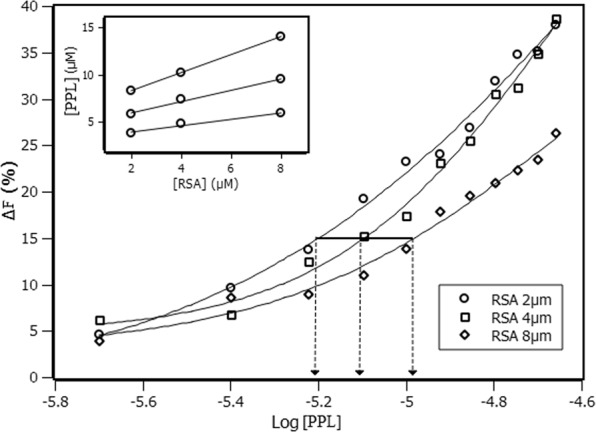
Percentage of quenching versus logarithm of PPL concentration with RSA at 2 µM, 4 µM and 8 µM. The horizontal solid line indicates the same percentage of quenching at different RSA concentrations. The inset shows the lines obtained for each set of quenching percentage.

Free ligand concentration and the average of interaction density are related to each other through the expression of mass conservation (Eq. (6)). By means of the graphic showed on Fig. 4, the values of [RSA] and [PPL] for each ΔF are obtained. The inset at Fig. 4 shows the graphics of [PPL] versus [RSA] for each ΔF, in which Σν_i_ is obtained from the slope, and [PPL]_free_ is obtained from y-intercept of the linear function.

6 $$[PPL]={[PPL]}_{free}+(\sum {\nu }_{i})\cdot [RSA]$$

**Figure 5:**
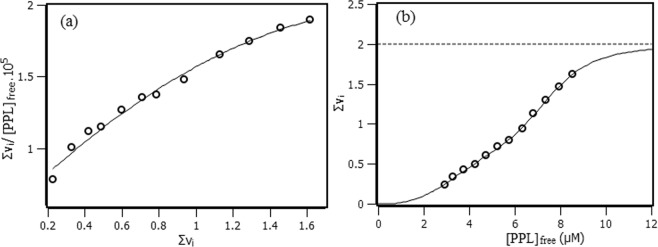
(**a**) Scatchard plot for the interaction of RSA and PPL obtained at 288 K based on IDF data. (**b**) Hill plot for the interaction of RSA and PPL obtained at 288 K based on IDF data.

Once the values of Σν_i_ and [PPL]_free_ were obtained from IDF method, Scatchard plot^20,21^ can reveal some aspects of RSA-PPL interaction. Figure 5a shows that Scatchard plot presents a convex curvature, which indicates that the system exhibited a positive cooperativity among the binding sites.

The number of sites and their respective binding constants were obtained by means of Hill equation^21^, which relates the average interaction density (Σν_i_) and the free ligand concentration [L_free_] through Eq. (7). Where n_j_ represents the number of sites of each set (j), k_j_ represents the binding constants for the sites, w_j_ the affinity indexes.

7 $${\sum }^{}{\nu }_{i}\,=\,\sum _{j}\frac{{n}_{j}{({k}_{j}[{L}_{free}])}^{{w}_{j}}}{1+\,{({k}_{j}[{L}_{free}])}^{{w}_{j}}}$$

Figure 5b shows the graphic of (Σν_i_) versus [PPL]_free_, where the solid line represents the best fit calculated with all the parameters explicit on Eq. (8).

8 $${\sum }^{}{\nu }_{i}=\,\frac{1{(2.3\times {10}^{5}[{L}_{free}])}^{2.93}}{1+\,{(2.3\times {10}^{5}[{L}_{free}])}^{2.93}}+\,\frac{1{(1.3\times {10}^{5}[{L}_{free}])}^{8.15}}{1+\,{(1.3\times {10}^{5}[{L}_{free}])}^{8.15}}$$

According to the results, PPL binds to 2 sites with different binding constants: k_1_ = (2.3 ± 0.1)10^5^ M^−1^ and k_2_ = (1.3 ± 0.1) 10^5^ M^−1^. Figure 5b presents a function profile typically of positive cooperativity between the sites of the macromolecule^22^. The binding constants obtained for this system are moderate constants with order of magnitude of 10^5^M^−1^. It is found in the literature^6^ a study of the interaction between PPL and human albumin, whose results are different from the present investigation in terms of the number of binding sites, affinity constant and thermodynamic balance. In this case, it is noticeable that the microenvironment of each protein is decisive to differentiate the interaction.

### Circular dichroism studies

**Figure 6:**
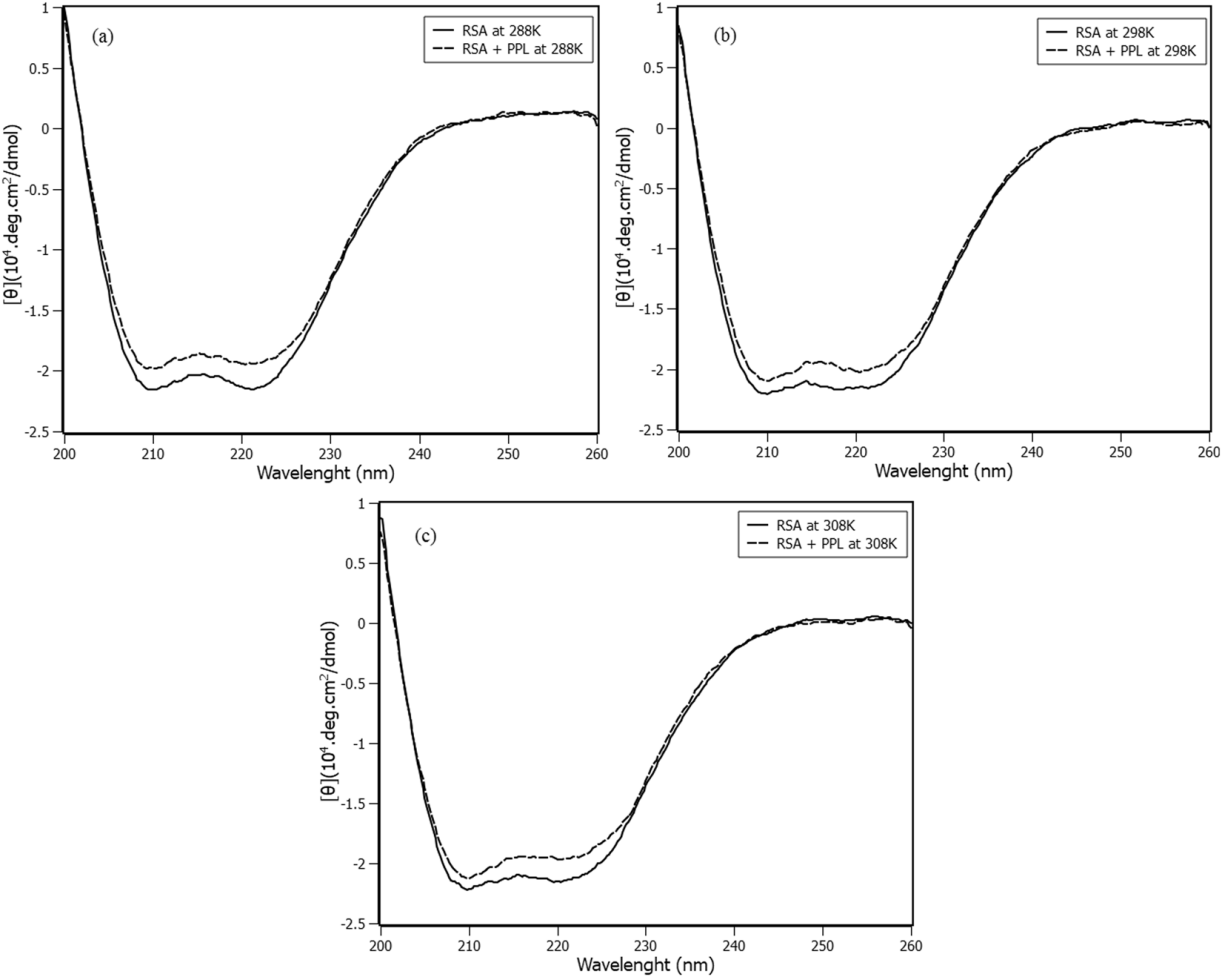
Circular Dichroism of free RSA (solid lines) and RSA with PPL at the stoichiometry 1:8 (dashed lines) at (**a**) 288K (**b**) 298K and (**c**) 308K.

Circular dichroism (CD) was carried out to follow the secondary structural changes of protein with the addition of PPL at different temperatures (Fig. 6).

Figure 6 (a → c) shows the CD spectra of RSA in the absence and presence of PPL at 288K, 298K and 308K, respectively. The CD spectra of free RSA at the three temperatures presented negative bands at 208 nm (π-π*) and 222 nm (n-π*), which are characteristics of α-helix content in the structure^22^. The calculated α-helix content remained very close to 63% even at different temperatures (Table S2). After PPL addition, the spectra kept the minimum at 208 nm and 222 nm, indicating that RSA native structure was preserved. The variation in the percentage of α-helix content was calculated, and represents only 2%, regardless of temperature. These results indicate that the secondary structure of RSA is preserved, regardless of the interaction with PPL and also the temperature. It was expected that the cooperative effect revealed by the IDF method was accompanied by some conformational change to configure the allosteric phenomenon. However, CD spectra show the absence of significant conformational changes in the RSA-PPL complex. Recent studies show that allosterism should not be necessarily accompanied by conformational changes^23^.

### Piperlongumine optimization by *ab initio* calculations

**Figure 7:**
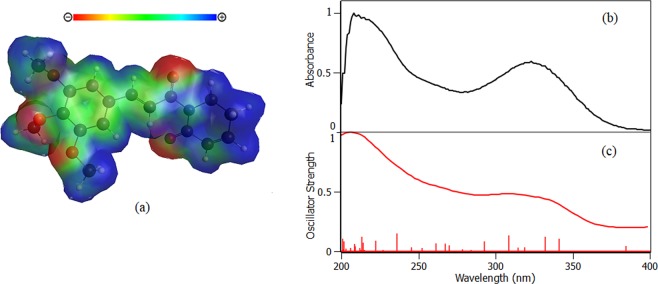
(**a**) Piperlongumine structure optimized by *ab initio* methods and the map of electrostatic potential (MEP). (**b**) Experimental absorbance spectrum of PPL in water. (**c**) Oscillator strength of PPL electronic transitions calculated by *ab initio* methods.

Before proceeding with molecular docking studies, PPL structure and partial charges were optimized by *ab initio* methods. The result of the structure optimization (Fig. 7a) showed a planar molecule with no torsion freedom for basically the entire structure except for methoxy groups. This result is due to the resonance of the electrons in the conjugated second order bonds of the aliphatic chain and the aromatic ring. Moreover, no imaginary frequency was found, which reveal that the structure found is not a structure in the transition state.

According to the map of electrostatic potential (MEP), the molecule has high negative charge density around the oxygen atoms and positive charge densities at the extremities of the molecule, while the scaffold is predominantly neutral. The oscillator strength (Fig. 7c) shows the main electrons transitions, which are in concordance with the experimental absorbance (Fig. 7b). The concordance between *ab initio* calculations and experimental results reinforced the structural optimization of PPL.

### Molecular docking

**Figure 8:**
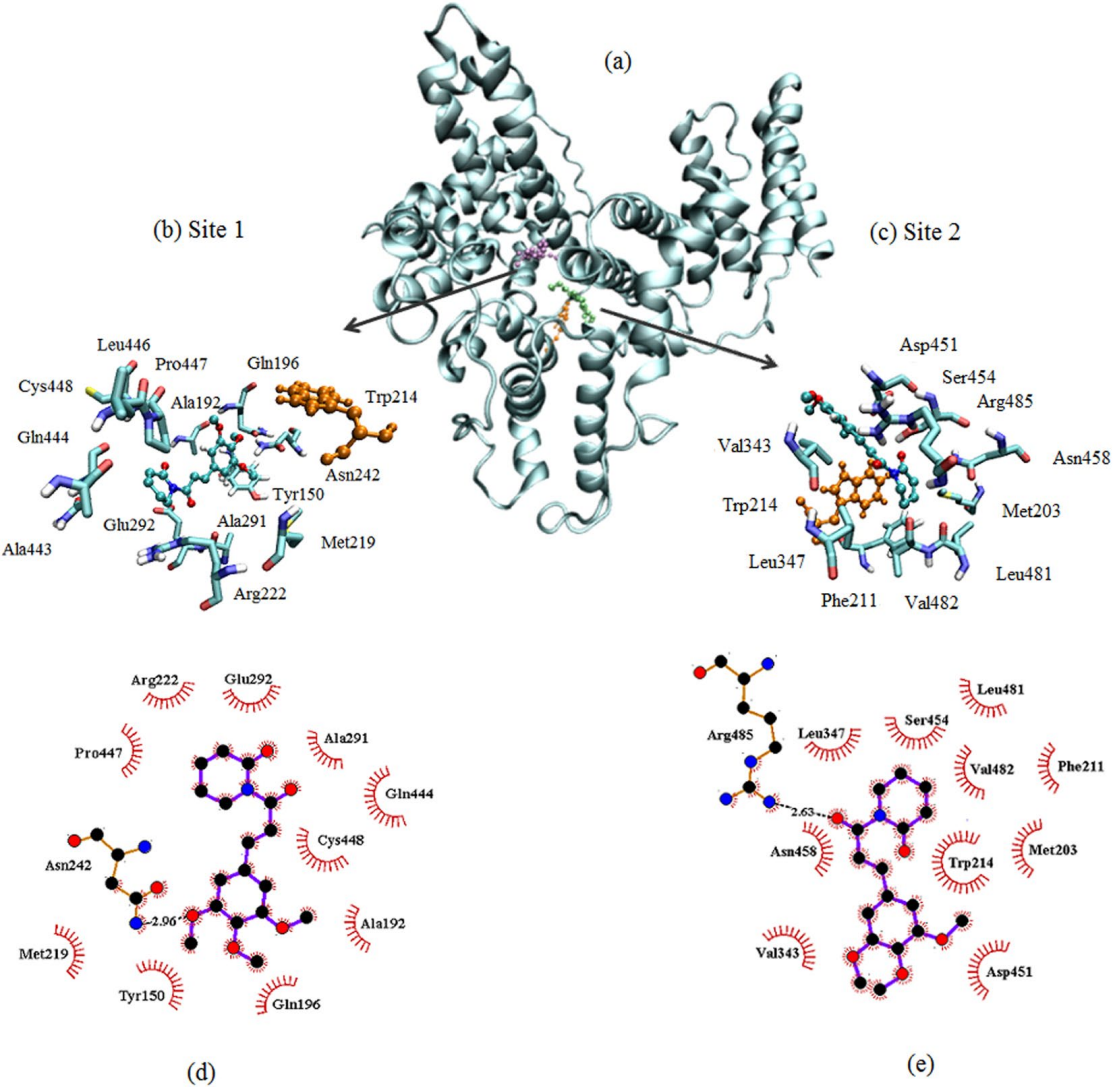
(**a**) Molecular docking with Trp214 in orange, PPL in site 1 (purple), and site 2 (green). Detail of the amino acids participating in the interaction at: (**b**) site 1, and (**c**) site 2. Interactions between amino acids and PPL: (**d**) Site 1, and (**e**) Site 2.

**Figure 9:**
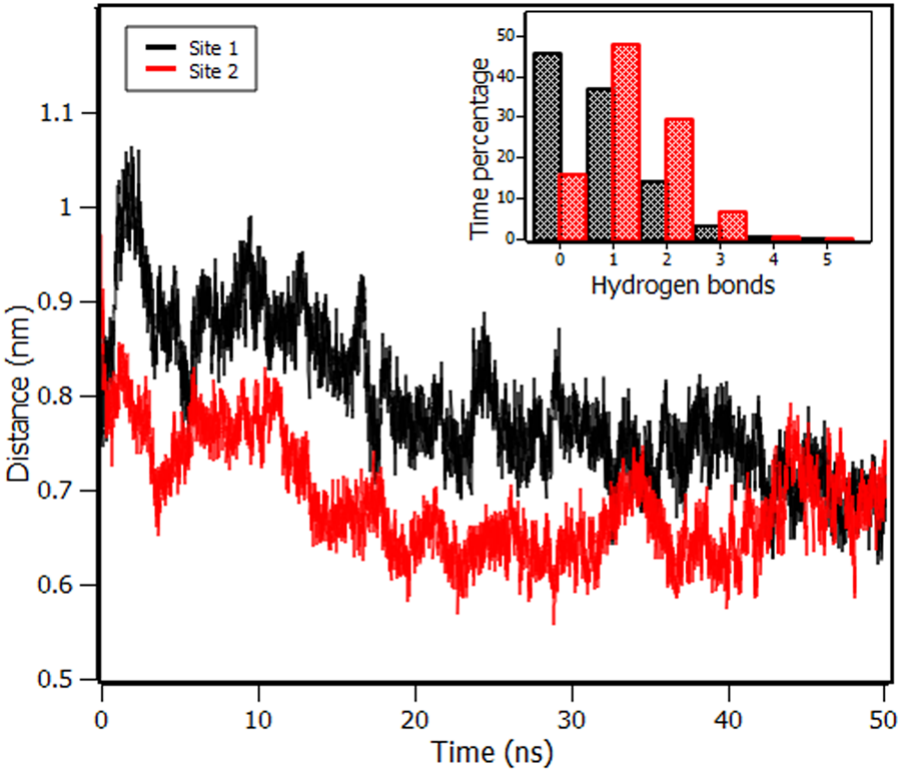
Distance between the centers of geometry (COG) of RSA and PPL in site 1 (black) and site 2 (red) along 50 ns of molecular dynamics. The inset shows the percentage of the time in which RSA and PPL makes different numbers of hydrogen bonds in site 1 (black) and in site 2 (red).

Molecular docking results (Fig. 8) revealed two sites with energy scores of − 4.75 and − 4.67 kcal/mol for site 1 and site 2, respectively. The sites are present in the microenvironment of Trp214 residue, which reinforces the results of fluorescence quenching process whose mechanism is static. Such mechanism requires some proximity between fluorophore(Trp214) and quencher (PPL) so that it is possible to occur the phenomenon of electron exchange known as Dexter interaction^13,24^. The number of sites is in agreement with IDF analyses. Site 1 is constituted by the apolar amino acids: Leu446, Met219, Trp214, Ala443, Ala291, Cys448, Pro447, Gln196, Gln444 and polar amino acids Glu292, Arg 222 and Asn242, such composition suggests that hydrophobic interactions are predominant (see Fig. 8d). Molecular docking also showed that for site 1, there is one hydrogen bond between Asn242 and PPL. Molecular dynamics (inset of Fig. 9) showed that the number of hydrogen bonds is no more than one in 83% of the time, even with the availability of the PPL molecule in performing up to five hydrogen bonds. These results indicated that hydrogen bonds do not play a major role in the complex stabilization. Similar characteristics are present at Site 2 which composition is of apolar amino acids: Trp214, Val343, Ser454, Leu347, Leu481, Val482, Phe211 and Met203 and polar amino acids: Arg 485, Asp451 and Asn 458. It is found one hydrogen bond between Arg485 and PPL. In this case, molecular dynamics (inset of Fig. 9) showed that the number of hydrogen bonds is no more than 1 in 64% of the time and therefore this kind of interaction also does not play the major role in the stabilization of PPL in site 2. The distances between COG of PPL and the amino acids present in Site 1 and 2 are presented in Table S3.

These findings are in agreement with the results obtained by Van’t Hoff analysis, which indicated that the major contribution of free energy is given by the entropic term and therefore, hydrophobic interactions drove the complex formation.

### Molecular dynamics

The root mean square deviation (RMSD) of RSA along 50 ns of molecular dynamics showed that the free protein remained stable after the first 20 ns of molecular dynamics, as well as the protein with PPL in Site 1 and Site 2(Fig. S6). The temporal stability of the complex formed by RSA and PPL was also verified through molecular dynamics simulations (Figs S7 and S8). Figure 9 shows that, after the first 20 ns, the distance between the centers of geometry (COG) of RSA and PPL fluctuated around 0.74 nm and 0.65 nm for sites 1 and 2, respectively.

The local stability of secondary structures around PPL in site 1 and 2 was investigated (Fig. S9) and compared to those obtained for free RSA. The subtle change in site 1 was the loss of α-helix character by the amino acids Arg222, Asp237 and Val238, which became part of turn content. Amino acids that compose binding site 2 presented high stability of the secondary structures, the pattern of the structures obtained for free RSA did not experience any significant changes when the MD was performed in the presence of PPL. The average percentage of the global secondary structures of the last 10 ns of the molecular dynamics was calculated for RSA at 298 K (Table S2). According to the results obtained, there was a decrease in the global α-helix content from 65% (free RSA) to 64% (PPL in both site 1 and 2). These results are in agreement with experimental data obtained by CD, which also showed a small decrease of the α-helix contents when PPL was added to the system.

## Conclusion

This work reveals the importance of the role of inner filter correction when dealing with fluorescence data. The approach performed through this correction results in the elimination of the shift of the spectrum that is commonly interpreted as a change in polarity in the fluorophore site. One of the novelties presented is the inclusion of the area of the emission spectrum band instead of the intensity, whose results are more complete taking into account that the emission curve simultaneously contains intensity, bandwidth and corrected baseline.

The RSA-PPL complex was characterized by a molecular biophysical approach with the fluorescence technique and computational methods. The thermodynamic balance from the experimental point of view indicated that the major energetic contribution came from entropy. This insight is corroborated by the result of molecular docking that confirms the largest number of apolar amino acids in the interaction sites.

Among the methods used to treat the fluorescence data, IDF showed to be more accurate, since in this method the formation of the complex meets the law of masses and therefore is not limited to a reaction of any order. IDF analyses together with Scatchard and Hill’s model brought up the most surprising result: the cooperative effect. This configures some allosteric mechanism in RSA, however protein allosterism in principle occurs in proteins with more than one subunit with conformational change. RSA is a monomeric protein that did not present conformational change when interacting with PPL. This result highlights the need for further studies to identify the routes by which sites communicate, and how the intrinsic dynamics of the protein is understood.

## Materials and Methods

### Reagents

Piperlongumine (>97%), RSA (>99%), Na_2_HPO_4_ (>99%), C_6_H_8_O_7_ (>99%), NaCl (>99%) were bought from Sigma-Aldrich (Schenelldorf, Bavaria, Germany). CH_3_OH was purchased from Dinâmica Química Contemporânea (Indaiatuba, SP, Brazil). Ultra filtered water was obtained from - Direct-Q UV-3 (MerckKgaA,Darmstadt, Germany). The preparation of the samples followed the previously described procedures^10^ and the concentration of the PPL stock solution was determined based on the molar extinction coefficient of 18700 M^−1^ cm^−1^ (at 326 nm).

### Steady-state fluorescence spectroscopy

#### Emission fluorescence

The equipment for measuring emission fluorescence was ISS PC1 (Champaign, IL, USA) with temperature controlled at 288, 298, 308 K, and the bandwidth set to 8 nm. The quartz cuvette has optical pathway of 10 mm. The sample was excited at 295 nm, and the emission spectrum was obtained in the region of 305–500 nm, with 10 reruns. The experiments of binding equilibrium were carried out with PPL increments of 2 μM. The baseline was corrected by subtracting the signal of the scattering of the buffer. The inner filter effects were rectified with Eq. 9, where F_corr_ and F_obs_ are the corrected and observed fluorescence intensities, and A_ex_ and A_em_ are the absorbance at the excitation wavelength (295 nm) and at emission wavelengths (from 305 to 500), respectively^13^.

9 $$\,{F}_{corr}={F}_{obs}\cdot {10}^{\frac{({A}_{ex}+{A}_{em})}{2}}$$

When adopting the approach of the areas, the numerical integration of each fluorescence spectrum was calculated from 305 to 400 nm in order to reduce the influence of PPL fluorescence signal. In the IDF analyses, PPL was titrated with increments of 2 μM at three different concentration of RSA (2 μM, 4 μM, and 8 μM) with temperature at 288 K. The final volume of methanol in the experiments did not influence the RSA fluorescence emission as verified in our previous work^10^.

#### Synchronous fluorescence

The equipment for measuring synchronous fluorescence was Lumina (Thermo Fisher Scientific, Waltham, MA, USA) with temperature controlled at 298 K, and the bandwidth set to 10 nm. The quartz cuvette has optical pathway of 10 mm. Excitation wavelength varied from 240 to 350 nm, the excitation and emission wavelengths interval (Δλ) was 60 nm, with 15 reruns. PPL was titrated with increments of 2 μM. The baseline was corrected by subtracting the signal of the scattering of the buffer. The inner filter effects were rectified with Eq. 9, where F_corr_ and F_obs_ are the corrected and observed fluorescence intensities, and A_ex_ and A_em_ are the absorbance at the excitation wavelengths (from 240 to 350 nm) and the emission wavelengths (from 300 to 410 nm), respectively.

#### Time-resolved fluorescence

The equipment for measuring fluorescence lifetime was Mini-τ coupled to a Time-Correlated Single Photon Counting (TCSPC) (Edinburgh Instruments, Livingston, UK). PPL was titrated varying the concentration from 0 to 32 μM, at 298 K. The excitation was done at 295 nm and the decay collected at 340 nm. The fluorescence decay profile (Supplementary Fig. S4) was fitted using multiexponential decay (Eq. 10), where τ_i_ is the lifetime of each component, and α_i_ is the contribution of each component to total fluorescence decay. The average lifetime τ_avg_ was calculated using Eq. 11 ^25^ (Table S1).

10 $${I}_{T}=\,\mathop{\sum }\limits_{i=1}^{n}{\alpha }_{i}\cdot {e}^{\frac{-T}{{\tau }_{i}}}$$

11 $$\,{\tau }_{avg}=\frac{{\alpha }_{1}{{\tau }_{1}}^{2}+{\alpha }_{2}{{\tau }_{2}}^{2}}{{\alpha }_{1}{\tau }_{1}+{\alpha }_{2}{\tau }_{2}}$$

#### Circular dischroism spectroscopy

CD experiments were carried out with Jasco J-815 (Jasco, Easton, MD, USA) at three different temperatures 288, 298, and 308 K, with a 0.01 cm optical pathway demountable quartz cell. The range of measurement was 200–260 nm with 15 reruns scanned at 20 nm/min and with 0.1 nm of resolution. The molar ratios employed for rat serum albumin and PPL were 1:0 and 1:8, with buffer spectrum subtracted. The ellipticity θ collected in millidegrees was converted to mean residue ellipticity [θ] (deg.cm^2^.dmol^−1^) using Eq. 12.

12 $$[\theta ]=\frac{\theta (mdeg)}{10.[P].l.n}$$

The secondary structures percentages were calculated with CDPro applying CONTIN method with SP43 protein library^26^.

#### Piperlongumine optimization by *ab initio* calculations

PPL structure optimization was calculated with Gamess2013^27,28^ with Hartree–Fock (HF) formalism^29^ and functional density theory (DFT)^30^ The first set of base 6–31 + G (d, p) was chosen which result was refined by the new set of bases 6–311 + G(2d, 2p), M11 functional and Polarizable Continuum Model (PCM) solvent model^31^ for water. The oscillator strength related to each electronic transition was calculated by TDDFT excite method with a set of bases 6–31 + G (d, p) and functional revTPSS. The geodesic method^32^ was applied to determine MEP and the partial charges. The facility of GRID UNESP was the computational center to perform all the calculations. The results visualized by wxMacMolPlt software^33^ and SciDavis (Free Software Foundation, Boston, MA, USA).

#### Molecular docking

All data obtained with respect to molecular docking followed the procedures previously described in our study^10^, except for grid box spacing of 0.436 Å, with a dimension of 126 × 126 × 126 points, and grid center coordinates of 59.236, 108.4, and 50.661 for x, y, and z coordinates, respectively. The final conformations were visualized by VMD^34^.

#### Molecular dynamics

The molecular dynamics of the complex were executed in triplicate by GROMACS/5.1.4^35^, with GROMOS96/53a force field^36^. Simple Point Charge(SPC) water model^37^ was used to solvate the complex, which was centered in a box with dodecahedral geometry. In order to neutralize the system, Na^+^ and Cl^−^ were added with 150 mM of concentration. A tolerance of 10 kJ/mol was set to the energy minimization step, through steepest descent algorithm. The NVT and NPT ensembles worked to reach the equilibrium along 100 ps of simulation where the temperature was maintained at 298 K by coupling to the V-rescale thermostat^38^ and the pressure was kept at 1 atm by coupling to the Parrinello–Rahman barostat^39^, respectively. Steps of 2 fs were set using the leap-frog algorithm to integrate the motion equations. The facility of GRID UNESP was the computational center to perform all the calculations. Hydrogen bonds were calculated through Visual Molecular Dynamics (VMD), OH and NH were regarded as donor, O and N were acceptors. The cut off for the angle hydrogen-donor-acceptor was 20 degrees and the cut off for the distance donor-acceptor was 0.3 nm^34^. The calculations of secondary structures of RSA were performed by VMD-TIMELINE plug-in employing STRIDE algorithm^40^.

## Supplementary information

Supplementary Information

## References

[1] Prasad S, K Tyagi A (2016). Historical spice as a future drug: therapeutic potential of piperlongumine. Curr. Pharm. Des..

[2] Bezerra DP (2013). Overview of the therapeutic potential of piplartine (piperlongumine). Eur. J. Pharm. Sci..

[3] Adams DJ (2012). Synthesis, cellular evaluation, and mechanism of action of piperlongumine analogs. Proc. Natl. Acad. Sci..

[4] Sun L-D (2015). Development and mechanism investigation of a new piperlongumine derivative as a potent anti-inflammatory agent. Biochem. Pharmacol..

[5] Zheng J (2016). Piperlongumine inhibits lung tumor growth via inhibition of nuclear factor kappa B signaling pathway. Sci. Rep..

[6] de Alcântara-Contessoto, N. S., Caruso, Í. P., Bezerra, D. P., Barbosa Filho, J. M. & Cornélio, M. L. An investigation into the interaction between piplartine (piperlongumine) and human serum albumin. *Spectrochim*. *Acta Part A Mol*. *Biomol*. *Spectrosc* (2019). 10.1016/j.saa.2019.04.076 31136859

[7] Liu Y (2018). Spectroscopic investigation of the anticancer alkaloid piperlongumine binding to human serum albumin from the viewpoint of drug delivery. Luminescence.

[8] Jagodzinski LL, Sargent TD, Yang M, Glackin C, Bonner J (1981). Sequence homology between RNAs encoding rat alpha-fetoprotein and rat serum albumin. Proc. Natl. Acad. Sci..

[9] Povinelli, A. P. R., Zazeri, G. & Cornélio, M. L. Molecular Mechanism of Flavonoids Using Fluorescence Spectroscopy and Computational Tools. In *Flavonoids-A Coloring Model For Cheering Up Life* (IntechOpen, 2019).

[10] Zazeri, G., Povinelli, P. A., Lima, D. M. & Cornélio, L. M. Experimental Approaches and Computational Modeling of Rat Serum Albumin and Its Interaction with Piperine. *International Journal of Molecular Sciences* **20** (2019). 10.3390/ijms20122856 PMC6627779 31212743

[11] He XM, Carter DC (1992). Atomic structure and chemistry of human serum albumin. Nature.

[12] Vivian JT, Callis PR (2001). Mechanisms of tryptophan fluorescence shifts in proteins. Biophys. J..

[13] Lakowicz Joseph R. (1999). Fluorescence Sensing. Principles of Fluorescence Spectroscopy.

[14] Lakowicz JR, Weber G (1973). Quenching of fluorescence by oxygen. Probe for structural fluctuations in macromolecules. Biochemistry.

[15] Soares S, Mateus N, De Freitas V (2007). Interaction of different polyphenols with Bovine Serum Albumin (BSA) and Human Salivary α-Amylase (HSA) by fluorescence quenching. J. Agric. Food Chem..

[16] Perrin, F. La fluorescence des solutions-Induction moléculaire.–Polarisation et durée d’émission.–Photochimie. in *Annales de physique* **10**, 169–275 (EDP Sciences, 1929).

[17] Bi S (2004). Investigation of the interaction between flavonoids and human serum albumin. J. Mol. Struct..

[18] Ross PD, Subramanian S (1981). Thermodynamics of protein association reactions: forces contributing to stability. Biochemistry.

[19] Lohman TM, Bujalowski W (1991). Thermodynamic methods for model-independent determination of equilibrium binding isotherms for protein-DNA interactions: spectroscopic approaches to monitor binding. Methods Enzymol..

[20] Scatchard G (1949). The attractions of proteins for small molecules and ions. Ann. N. Y. Acad. Sci..

[21] Bordbar AK, Saboury AA, Moosavi‐Movahedi AA (1996). The shapes of Scatchard plots for systems with two sets of binding sites. Biochem. Educ..

[22] Van Holde, K. E., Johnson, W. C. & Ho, P. S. Principles of physical biochemistry. (2006).

[23] Liu, J. & Nussinov, R. Allostery: an overview of its history, concepts, methods, and applications. (2016). 10.1371/journal.pcbi.1004966 PMC4890769 27253437

[24] Hassoon S, Lustig H, Rubin MB, Speiser S (1984). The mechanism of short-range intramolecular electronic energy transfer in bichromophoric molecules. J. Phys. Chem..

[25] Zaidi N (2013). Biophysical insight into furosemide binding to human serum albumin: a study to unveil its impaired albumin binding in uremia. J. Phys. Chem. B.

[26] Sreerama N, Woody RW (2000). Estimation of protein secondary structure from circular dichroism spectra: comparison of CONTIN, SELCON, and CDSSTR methods with an expanded reference set. Anal. Biochem..

[27] Schmidt MW (1993). General atomic and molecular electronic structure system. J. Comput. Chem..

[28] Gordon, M. S. & Schmidt, M. W. Advances in electronic structure theory: GAMESS a decade later. In *Theory and applications of computational chemistry* 1167–1189 (Elsevier, 2005).

[29] Slater JC (1951). A simplification of the Hartree-Fock method. Phys. Rev..

[30] Parr, R. G. Density functional theory of atoms and molecules. In *Horizons of Quantum Chemistry* 5–15 (Springer, 1980).

[31] Tomasi J, Mennucci B, Cammi R (2005). Quantum mechanical continuum solvation models. Chem. Rev..

[32] Spackman MA (1996). Potential derived charges using a geodesic point selection scheme. J. Comput. Chem..

[33] Bode BM, Gordon MS (1998). MacMolPlt: a graphical user interface for GAMESS. J. Mol. Graph. Model..

[34] Humphrey W, Dalke A, Schulten K (1996). VMD: visual molecular dynamics. J. Mol. Graph..

[35] Van Der Spoel D (2005). GROMACS: fast, flexible, and free. J. Comput. Chem..

[36] Oostenbrink C, Villa A, Mark AE, Van Gunsteren WF (2004). A biomolecular force field based on the free enthalpy of hydration and solvation: the GROMOS force‐field parameter sets 53A5 and 53A6. J. Comput. Chem..

[37] Berendsen, H. J. C., Postma, J. P. M., van Gunsteren, W. F. & Hermans, J. Interaction models for water in relation to protein hydration. In *Intermolecular forces* 331–342 (Springer, 1981).

[38] Bussi G, Donadio D, Parrinello M (2007). Canonical sampling through velocity rescaling. J. Chem. Phys..

[39] Parrinello M, Rahman A (1981). Polymorphic transitions in single crystals: A new molecular dynamics method. J. Appl. Phys..

[40] Frishman D, Argos P (1995). Knowledge‐based protein secondary structure assignment. Proteins Struct. Funct. Bioinforma..

