# Supplementary material for: Details of the cooperative binding of piperlongumine with rat serum albumin obtained by spectroscopic and computational analyses
Source: Sci Rep. 2019 Oct 30;9:15667. doi: 10.1038/s41598-019-52187-5 (PMC6821919; doi:10.1038/s41598-019-52187-5)
Supplement: Supplementary file 1 — Supplementary Information [file 41598_2019_52187_MOESM1_ESM.pdf]

Supplementary material

Details of the cooperative binding of piperlongumine with rat serum albumin obtained by spectroscopic and computational analyses

Ana Paula Ribeiro Povinelli[1†],Gabriel Zazeri[1†], Marcelo de Freitas Lima[2] and Marinônio Lopes Cornélio [1,*]

[1] Departamento de Física, Instituto de Biociências, Letras e Ciências Exatas (IBILCE), UNESP, Rua Cristovão Colombo 2265, CEP 15054-000, São José do Rio Preto, SP, Brazil; ana.povinelli@unesp.br (A.P.R.P.), gabriel.zazeri@unesp.br (G.Z.)
[2] Departamento de Química, Instituto de Biociências, Letras e Ciências Exatas (IBILCE), UNESP, Rua Cristovão Colombo 2265, CEP 15054-000, São José do Rio Preto, SP, Brazil. marcelo.f.lima@unesp.br
[*] Correspondence: m.cornelio@unesp.br
† These authors contributed equally to this work

```
HSA DAHKSEVAHRFKDLGEENFKALVLIAFAQYLQQCPFEDHVKLVNEVTEFAKTCVADESAE  60
RSA EAHKSEIAHRFKDLGEQHFKGLVLIAFSQYLQKCPYEEHIKLVQEVTDFAKTCVADENAE  60
    :*****:*********::**.******:****:**:*:*:***:***:*********.**

HSA NCDKSLHTLFGDKLCTVATLRETYGEMADCCAKQEPERNECFLQHKDDNPNLPRLVRPEV 120
RSA NCDKSIHTLFGDKLCAIPKLRDNYGELADCCAKQEPERNECFLQHKDDNPNLPPFQRPEA 120
    *****:*********:: .**:.***:************************** : ***.

HSA DVMCTAFHDNEETFLKKYLYEIARRHPYFYAPELLFFAKRYKAAFTECCQAADKAACLLP 180
RSA EAMCTSFQENPTSFLGHYLHEVARRHPYFYAPELLYYAEKYNEVLTQCCTESDKAACLTP 180
    :.***:*::*  :** :**:*:*************::*::*: .:*:**  :****** *

HSA KLDELRDEGKASSAKQRLKCASLQKFGERAFKAWAVARLSQRFPKAEFAEVSKLVTDLTK 240
RSA KLDAVKEKALVAAVRQRMKCSSMQRFGERAFKAWAVARMSQRFPNAEFAEITKLATDVTK 240
    *** ::::. .::.:**:**:*:*:*************:*****:*****::**.**:**

HSA VHTECCHGDLLECADDRADLAKYICENQDSISSKLKECCEKPLLEKSHCIAEVENDEMPA 300
RSA INKECCHGDLLECADDRAELAKYMCENQATISSKLQACCDKPVLQKSQCLAEIEHDNIPA 300
    ::.***************:****:**** :*****: **:**:*:**:*:**:*:*::**

HSA DLPSLAADFVESKDVCKNYAEAKDVFLGMFLYEYARRHPDYSVVLLLRLAKTYETTLEKC 360
RSA DLPSIAADFVEDKEVCKNYAEAKDVFLGTFLYEYSRRHPDYSVSLLLRLAKKYEATLEKC 360
    ****:******.*:************** *****:******** *******.**:*****

HSA CAAADPHECYAKVFDEFKPLVEEPQNLIKQNCELFEQLGEYKFQNALLVRYTKKVPQVST 420
RSA CAEGDPPACYGTVLAEFQPLVEEPKNLVKTNCELYEKLGEYGFQNAVLVRYTQKAPQVST 420
    ** .**  **..*: **:******:**:* ****:*:**** ****:*****:*.*****

HSA PTLVEVSRNLGKVGSKCCKHPEAKRMPCAEDYLSVVLNQLCVLHEKTPVSDRVTKCCTES 480
RSA PTLVEAARNLGRVGTKCCTLPEAQRLPCVEDYLSAILNRLCVLHEKTPVSEKVTKCCSGS 480
    *****.:****:**:***. ***:*:**.*****.:**:***********::*****: *

HSA LVNRRPCFSALEVDETYVPKEFNAETFTFHADICTLSEKERQIKKQTALVELVKHKPKAT 540
RSA LVERRPCFSALTVDETYVPKEFKAETFTFHSDICTLPDKEKQIKKQTALAELVKHKPKAT 540
    **:******** **********:*******:***** :**:********.**********

HSA KEQLKAVMDDFAAFVEKCCKADDKETCFAEEGKKLVAASQAALGL                585
RSA EDQLKTVMGDFAQFVDKCCKAADKDNCFATEGPNLVARSKEALA-                584
    ::***:**.*** **:***** **:.*** ** :*** *: **.
```

Figure S1: Sequence alignment of Human Serum Albumin (HSA) and Rat Serum Albumin (RSA) with 73% of similarity. Asterisk indicates positions which have a single, fully conserved residue. Colon indicates conservation between groups of strongly similar properties. Period indicates conservation between groups of weakly similar properties.

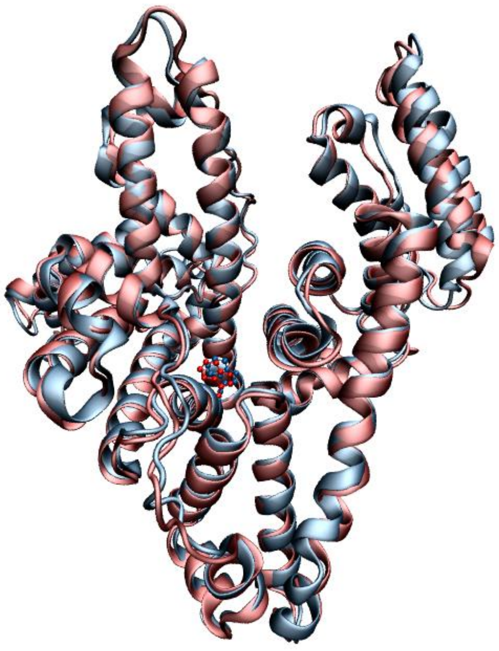

Figure S2: Structural alignment of RSA (blue) and HSA-PDB 1AO6 (red). In "ball and stick" style TRP214 of RSA (blue) and HSA (red).

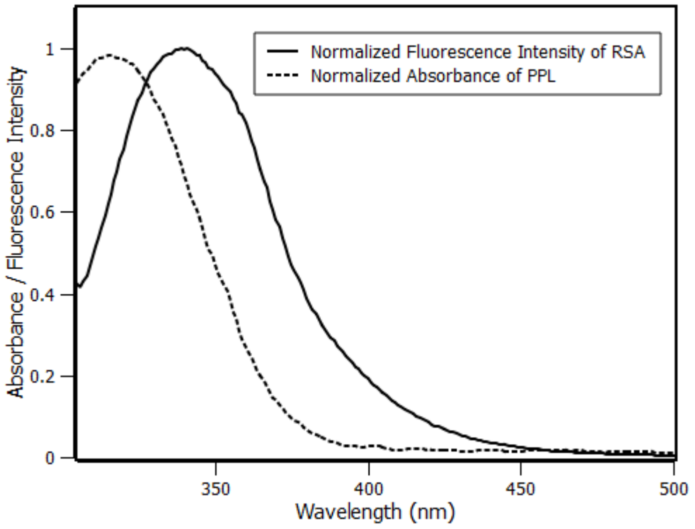

Figure S3: Spectra of absorption of PPL and emission of RSA in the range from 305 to 500 nm.

Time-dependent fluorescence decays (Figure S4) was fitted using multiexponencial decay (Equation 10). The best fit was obtained with two lifetimes τ_1 and τ_2 (Table S1). The average time (τ_{avg}) was calculated by Equation 11 considering the contributions, α_1 and α_2, of each component.

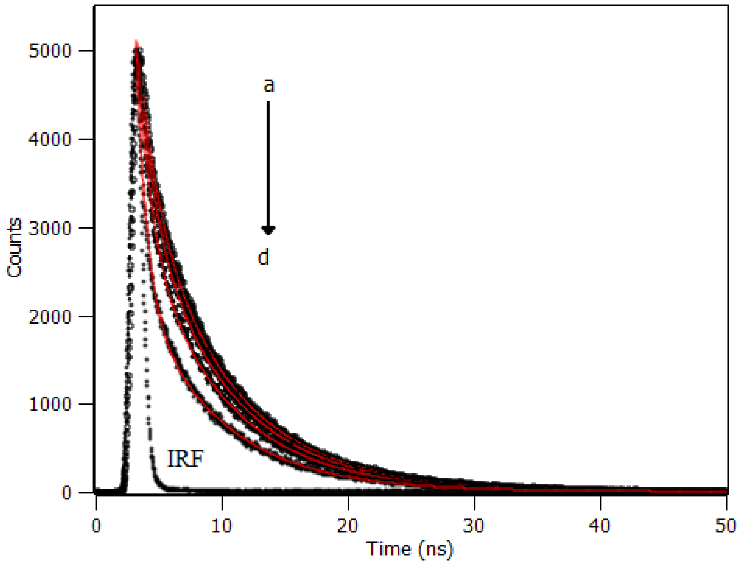

Figure S4: Time-dependent fluorescence decay of (a) RSA and (→d) in the RSA:PPL stoichiometries 1:1, 1:3, 1:8. [RSA] = 4μM, T = 298K and λ_{exc} = 295nm. IRF indicates the instrument response function and the red solid line is the biexponential fitting for each decay.

Table S1: Tryptophan lifetime in different stoichiometries RSA:PPL obtained through biexponential decay.

RSA : PPL	α_1	τ_1 (ns)	α_2	τ_2 (ns)	τ_{avg} (ns)	χ^2
**1:0**	0.08	1.51	0.92	6.81	6.71	1.10
**1:1**	0.09	1.33	0.91	6.74	6.64	1.12
**1:3**	0.10	1.11	0.90	6.60	6.50	1.16
**1:8**	0.13	0.81	0.86	6.39	6.28	1.20

The binding constants were calculated through the linear adjustments of the double-log plots.

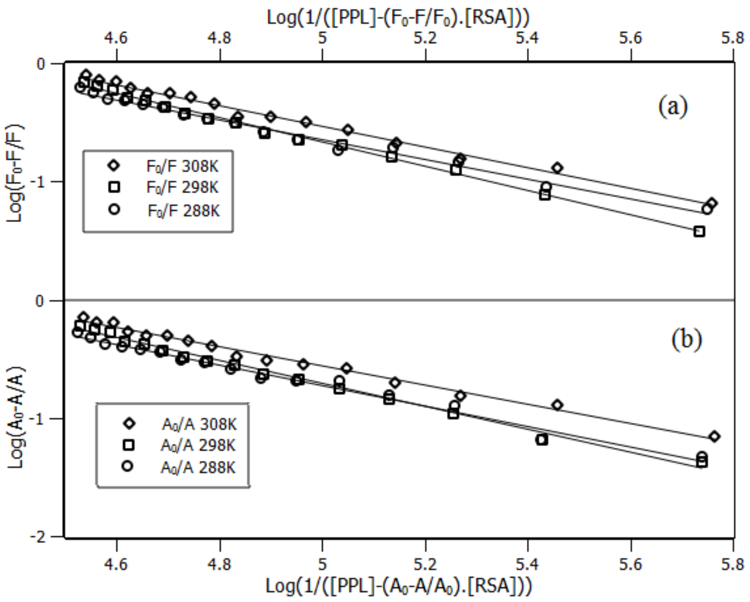

Figure S5: Double-log plots using (a) the fluorescence intensity at 340 nm and (b) the areas below the spectra (from 305 to 400 nm).

Table S2: Secondary Structures percentages obtained experimentally from Circular Dichroism (CD) and obtained computationally from Molecular Dynamics (MD). Circular Dichroism experiments were carried out at the stoichiometry RSA:PPL (1:8) at 288K, 298K and 308K. Molecular Dynamics were performed for RSA, RSA+PPL in site 1 (MD 1) and RSA+PPL in site 2 (MD 2) at 298K .

Sample	α-helix(%)	Turns(%)	Random Coil(%)
**RSA 288K – CD**	63	17	16
**RSA 298K – CD**	63	17	17
**RSA 298K – MD**	65	17	16
**RSA 308K – CD**	62	17	19
**RSA+PPL 288K – CD**	61	19	15
**RSA+PPL 298K – CD**	61	18	18
**RSA+PPL 298K – MD 1**	64	19	17
**RSA+PPL 298K – MD 2**	64	16	19
**RSA+PPL 308K – CD**	60	19	18

The stability of RSA structure along 50 ns of molecular dynamics was verified by means of the root mean square deviation (RMSD) with free RSA, PPL in site 1 and PPL in site 2.

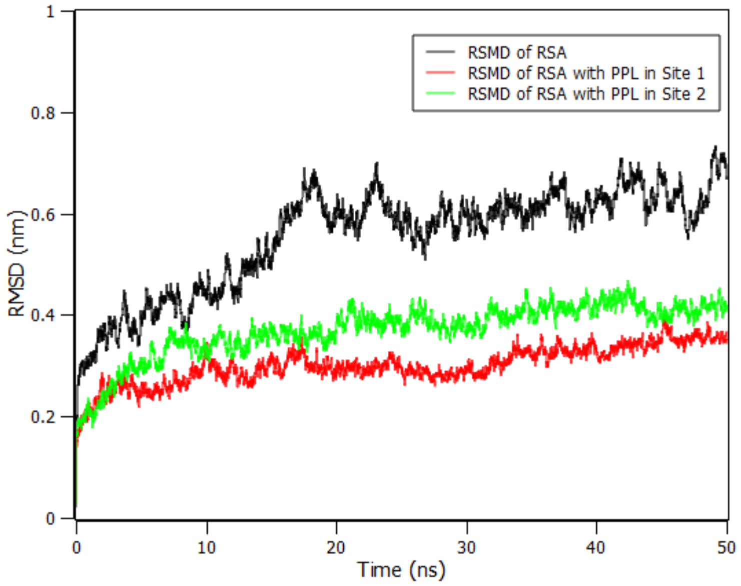

Figure S6: Root mean square deviation (RMSD) calculated for the backbone of free RSA(black), RSA with PPL in site 1 (red) and RSA with PPL in site 2 (green).

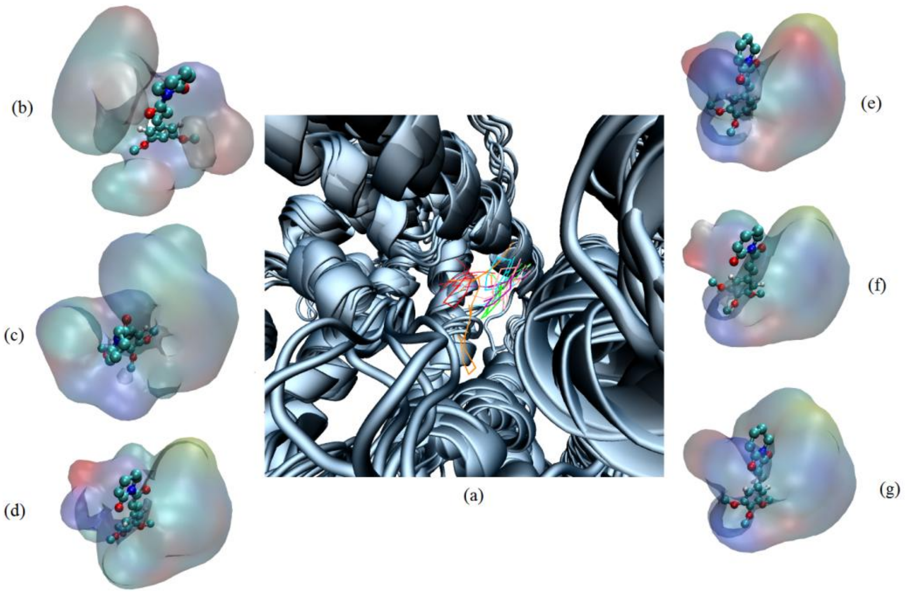

Figure S7 : (a) Structural conformation of the complex RSA-PPL, at site 1, from 0 to 50 ns, with intervals of 10 ns. (b → g) conformational configuration of the RSA-PPL microenvironment (site 1) with the participation of amino acids (quicksurf) at a distance of 8 Å, during 50ns at 10ns intervals.

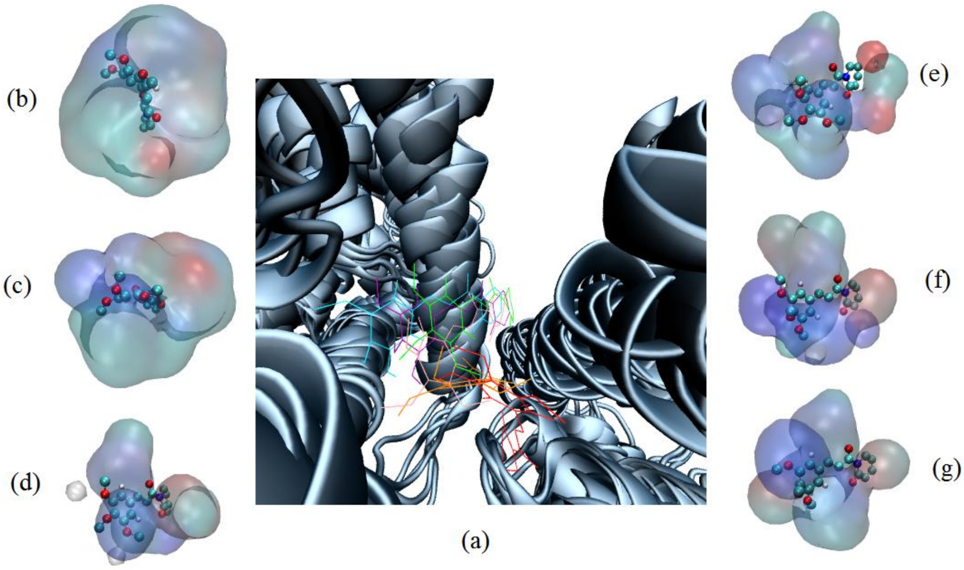

Figure S8 : (a) Structural conformation of the complex RSA-PPL, at site 2, from 0 to 50 ns, with intervals of 10 ns. (b → g) conformational configuration of the RSA-PPL microenvironment (site 2) with the participation of amino acids (quicksurf) at a distance of 8 Å, during 50ns at 10ns intervals.

Molecular dynamics analyses also showed the stability of secondary structures present in site 1 and site 2 microenvironment.

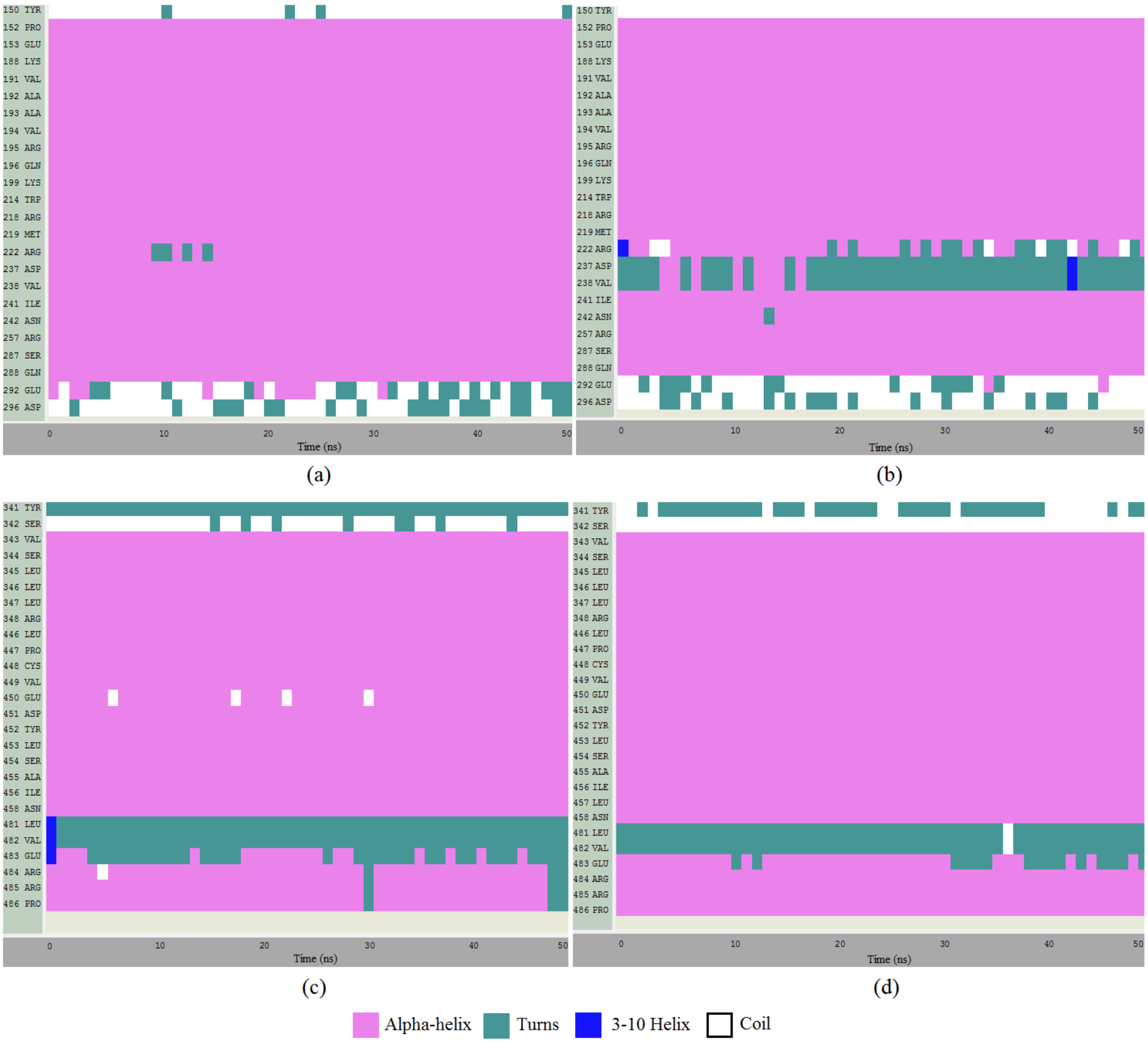

Figure S9: Secondary structure of amino acids that compose site 1 during 50 ns of MD: (a) free RSA, (b) RSA with PPL. Secondary structure of amino acids that compose site 2 during the 50 ns of MD: (c) free RSA and (d) RSA with PPL.

Table S3: Distance between COG of PPL and COG of the amino acid residues that compose the microenvironment of Site 1 and Site 2 in Molecular Docking.

**Site 1**		**Site 2**	
**Residue**	**Distance (nm)**	**Residue**	**Distance (nm)**
Trp214	1.18	Trp214	0.50
Cys448	1.03	Asn458	0.99
Leu446	1.35	Leu481	1.14
Arg222	1.02	Ser454	0.58
Ala291	0.77	Arg485	0.71
Ala443	1.47	Leu347	0.86
Gln444	1.22	Val343	0.54
Glu292	0.69	Phe211	0.98
Met219	1.05	Val482	0.94
Pro447	0.90	Met203	1.22
Ala192	0.85	Asp 451	0.47
Gln196	1.01	-	-
Asn242	0.92	-	-
Tyr150	0.89	-	-

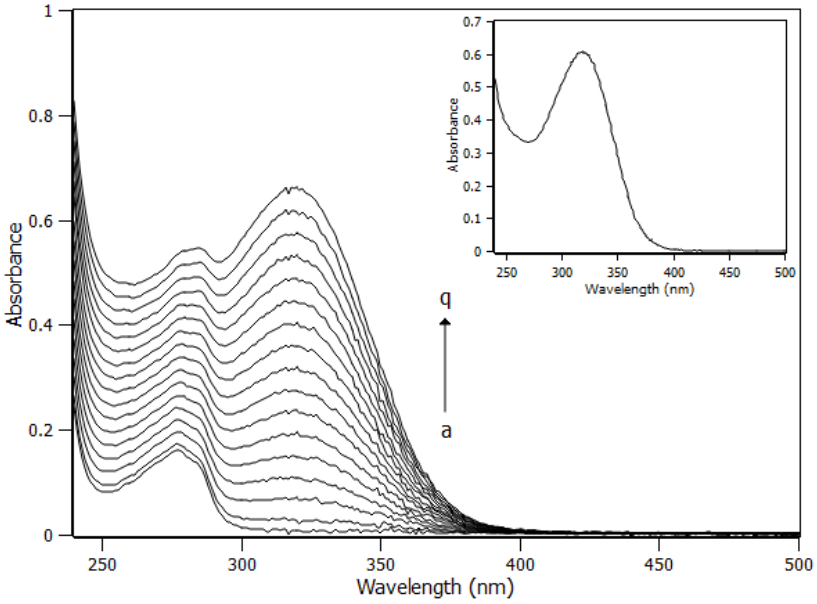

Figure S10: UV-Vis spectra of RSA with increments of 2µM of PPL (a→q: from 0 to 32µM), [RSA] = 4µM. The inset shows the UV-Vis spectrum of PPL at 32µM in buffer.
